# Language modulates brain activity underlying representation of kinship terms

**DOI:** 10.1038/srep18473

**Published:** 2015-12-21

**Authors:** Haiyan Wu, Yue Ge, Honghong Tang, Yue-Jia Luo, Xiaoqin Mai, Chao Liu

**Affiliations:** 1 Key Laboratory of Behavioral Science, Institute of Psychology, Chinese Academy of Sciences, 100101, Beijing, China; 2 State Key Laboratory of Cognitive Neuroscience and Learning & IDG/McGovern Institute for Brain Research, Beijing Normal University, Beijing, 100875, China; 3 Center for Collaboration and Innovation in Brain and Learning Sciences, Beijing Normal University, Beijing, 100875, China; 4 Beijing Institute of Biomedicine, Beijing, China; 5 Institute of Affective and Social Neuroscience, Shenzhen University, Shenzhen, Guangdong; 6 Department of Psychology, Renmin University of China, Beijing, 100872, China

## Abstract

Kinship terms have been found to be highly diverse across languages. Here we investigated the brain representation of kinship terms in two distinct populations, native Chinese and Caucasian English speakers, with a five-element kinship identification (FEKI) task. The neuroimaging results showed a common extensive frontal and parietal lobe brain activation pattern for different kinship levels for both Chinese and Caucasian English speakers. Furthermore, Chinese speakers had longer reaction times and elicited more fronto-parietal brain networks activation compared to English speakers in level three (e.g., uncle and nephew) and four (e.g., cousin), including an association between the middle frontal gyrus and superior parietal lobe, which might be associated with higher working memory, attention control, and social distance representation load in Chinese kinship system processing. These results contribute to our understanding of the representation of kinship terms in the two languages.

Kinship is one of the closest bonds between people and plays a key role in many aspects of social life, including the rules of inheritance, parental investment, and prosocial behaviors^1^. Although kinship is a fundamental clue to define interpersonal relationships, the functional structure of kinship representation in the mind remains unclear. Researchers have proposed that kinship representation can be analogous to the spatial representation of the physical space (Jones, 2010), such that “*Kinship maps always utilize spatial imagery. I have seen no exception to this. People around the world commonly speak of “sides”, “lines”, “distant” or “close” relatives, and reckon relationships “upward” or “downward”*. *Because of this, the best way to capture the conceptual structure of systems of kin definitions without using one’s own cultural conceptions as an obscuring filter is usually to ask for diagrams, not lists”*^2^^ p. 308^.

A practical way to study such a spatial representation of kinship is through kinship terms, such as “father”, “brothers”, and “sisters”. These terms are usually studied in the field of anthropology, but they can serve as a basis to infer the cognitive representation of kinship. For example, Romney *et al.*^3^ selected 15 kinship terms in English and conducted a semantic structure analysis. The results showed that semantic structure was consistent with genetic relatedness.

To our knowledge, no study has directly examined the neural bases of kin terms representation under the framework of kinship concept structure, assessed neural network activation for different kinship levels, or studied the influence of language on kinship representation. Most previous neuroimaging studies of kinship focused on kinship identification, i.e. the process by which kinship is identified using external cues such as facial-resemblance^4^,^5^,^6^,^7^, olfactory stimuli^8^, even names^9^ or family faces^10^,^11^. For example, Lundstrom *et al.*^12^ found that olfactory-based kin recognition activated the dorsal-medial prefrontal cortex, frontal-temporal junction, and insula. In contrast, when viewing their newborn infants’ photos, mothers recruited the bilateral orbitofrontal cortex (OFC) and occipital cortex to a greater extent than when they viewed photos of unfamiliar infants^13^. A similar study reported amygdala and superior temporal sulcus (STS) activation when mothers viewed their own children versus other children^14^.

Although kin term systems are mainly derived from genetic relatedness within a population, they can also be affected by external factors such as constraints in the kinship domain across different languages^15^. The cross-language perspective of kinship term representation is particularly interesting given the well-known kin terms system differences across language. To avoid cognitive complexity, the kin term system requires a trade-off between simplicity and communicative precision. For example, kin terms in English seem relatively simple, whereas some eastern languages adopt more constraints to maintain categorical precision^16^. Two representations of simple and complex kin term system are English and Chinese. There are 15 kin terms in English, while there are 33 kin terms in Chinese. As described by Alfred L. Kroeber, “*The Chinese obviously remain interested in kinship, whereas we want to refer to it as sketchily as possible. To use another simile, they are like people who want to know the exact time, we like those who would rather estimate by the sun than be bothered to keep clocks running”*^17^^ p. 156^. To explore the constraints underlying the conceptual structure of Chinese kinship, Liu *et al.*^18^ developed a five-element kinship identification (FEKI) task and conducted a behavioral study in Chinese subjects. This study showed that kinship structure was governed by several constraints including distance, generations, sex, and relative age.

In the current study, we performed an fMRI study of English and Chinese speakers to determine whether there is an interaction between kinship constraints and language in the representation of kinship terms. According to the kinship distance of kin terms, we examined kinship representation in English versus Chinese speakers for four kinship levels: 1) the first level (lv1) represents kin of the closest distance, including parents, offspring, and siblings; 2) the second level (lv2) represents the two-distance lineal kin, such as grandparent (e.g., father’s father) and grandchildren (e.g., son’s son); 3) the third level (lv3) represents the two-distance collateral kin, such as uncle, nephew, and niece; 4) the fourth level (lv4) represents distant kin (e.g., cousins). We expected to identify brain activities associated with spatial processing (e.g., distance processing) to be involved in kinship level processing for both languages, especially for Chinese subjects given the more complicated kinship level evaluation of Chinese kin terms.

## Method

### Participants

Twenty native Chinese-speaking Chinese subjects (10 males, *M* = 22 years, *SD* = 1.6 years) and twenty native English-speaking Caucasian subjects (12 males, *M* = 23 years, *SD* = 5.6 years) participated in the present study, for which they were compensated. All the native English-speaking subjects were foreigners living in Beijing (less than two years). All subjects were right-handed with normal or corrected-to-normal vision. To reduce the influence of the one-child policy in Chinese participants, all participants were screened a priori to ensure that they had at least one sibling. Therefore, the number of lv1 relatives was not significantly different between the Chinese (*M* = 3.35, *SE* = 0.19) and Caucasian Group (*M* = 3.45, *SE* = 0.19). The study was carried out in accordance with the Declaration of Helsinki and the experimental protocols used were approved by by the institutional review board (IRB) of Beijing Normal University. Informed written consent was obtained from each participant.

### Procedure

**Figure 1:**
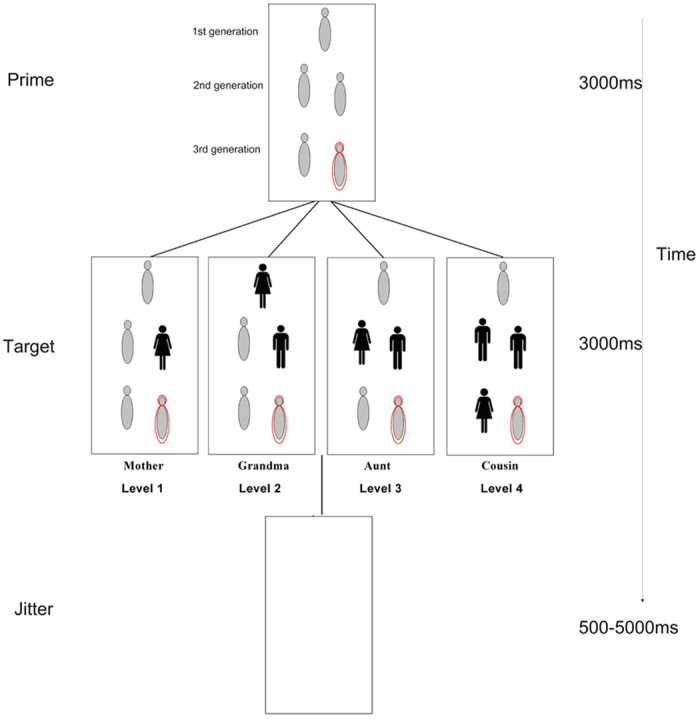
Sample stimuli from the experimental stimuli set and the procedure of each trial: Each trial begins with a prime representing the subject in the kinship space displayed for 3000 ms, then a target or several male or female target units presented for 3000 ms spaced by three inter-phase intervals which varied between 500–6500 ms. Participants were asked to report the kin term of the farthest target in the kinship space and press the spacebar at the same time.

We employed the FEKI task described by Liu *et al.*^18^ (see Fig. 1). In the “kinship space” of the FEKI task, all possible kinship terms over three generations are categorized into 5 elements and distinguished by sex, generations, distance, and relative age in a single schema. There is one element in the first generation (the top row) that comprises the grandparent unit. The second generation (the middle row) represents the offspring of the grandparents and comprises two elements: a sibling unit and a parental unit. The third generation (the bottom row) corresponds to the offspring of the second generation and comprises two elements: a cousin unit and a child unit. To account for age within generations, the uppermost position in each row represents the oldest member of each generation, while the lower position represents the youngest. Finally, to distinguish sex and patrikin, each unit is portrayed with widely used symbols for males (with pants) versus females (with skirts). Using this schema, it is thereby possible to represent the 4 levels of kinship distance in one diagram, while also accounting for generation (row), age (position within a row), and sex/patrikin (male versus female symbols). Moreover, in this schema, all kinship representations present in English and Chinese are diagrammed, allowing for cross- language assessment.

Experimentally, participants can be asked to express the relationship of a single specified unit (the prime unit) to other units (target units) with largest kinship distance in the schema. Kinship level processing can be assessed in this task by measuring the reaction time necessary to distinguish kinship relationships, which is thought to correlate with kinship expression complexity. To assess brain activation patterns associated with kinship activation, such tasks can be conducted in conjunction with functional magnetic resonance imaging (fMRI).

In the experiment used in this study, participants were asked to identify the relationship between a prime unit (indicated by a red circle outline) and five randomly selected target units for 3000 ms. Specifically, the task was to report the kinship term representing the relationship between the prime unit and the farthest target unit while simultaneously pressing a key. Chinese and English subjects reported the terms in their native language. Trials were separated by a jitter ranging from 500 to 6000 ms. Before the fMRI session, there was a short practice session to make sure each participant understood the paradigm and the key press rule (i.e., orally report the kin term and press the spacebar at the same time). The fMRI session was composed of 4 runs with 396 trials in total (with an identical number of trials for each kinship level). After the fMRI scan, participants were asked to describe kinship in their own lives in a full kinship list and rate the corresponding intimacy with a 7 point scale.

### Image data acquisition

The fMRI scanning was conducted in a 3-T Siemens MRI scanner (Siemens, Erlangen, Germany) in the MR center of Beijing Normal University, Beijing, China. Functional images were collected transversely by using an echo-planar imaging (EPI) sequence with the following parameters: TR = 1500 ms, TE = 28 ms, flip angle = 75°, FOV = 200 mm, slices = 28, in-plane matrix = 64 × 64, thickness = 3 mm, interslice gap = 0.2 mm, voxel size = 3.1 × 3.1 × 3.1 mm^3^. Three-dimensional T1-weighted anatomical images were collected axially using a 3-D spoiled gradient recalled (SPGR) sequence (TR = 2300 ms, TE = 2.98 ms, flip angle = 9°, slice thickness = 1 mm, FOV = 24 × 24 cm^2^, matrix size = 512 × 512 × 170, and voxel size = 0.5 × 0.5 × 1 mm^3^) on each subject.

### Data analysis

Data were preprocessed and analyzed using SPM5 (Wellcome Department of Imaging Neuroscience, Institute of Neurology, London; http://www.fil.ion.ucl.ac.uk/spm), implemented in Matlab (Mathworks, USA). The first two scans were excluded from the analysis to eliminate nonequilibrium effects of magnetization. The time series for each voxel was interpolated to correct for nonsimultaneous slice acquisition within each volume. Slice timing was corrected using the middle slice as the reference. The mean of all images for each participant was normalized to Montreal Neurological Institute space^19^. We smoothed the brain images with a 8-mm full-width/half-maximum kernel. Images were filtered (high-pass filter set at 128 s, low-pass filter achieved by convolution with hemodynamic response function). The resulting images had cubic voxels of 2 × 2 × 2 mm^3^.

After preprocessing, statistical analysis was carried out using the general linear model^19^ for the underlying neural response to four experimental conditions (kinship lv1 to lv4) in the two groups, all of which were time-locked to the onset time of the presentation of the target. The four trial types were modeled as separate regressors and interrogated to derive contrast images for second-level analysis. We first performed a group-level random effects analysis by conducting a one-sample t-test across all individual participants and examined the activation for each kinship level with a threshold of *P*_FWE_ < 0.005, extant threshold >30 voxels.

To examine our hypotheses that there would be language differences, we performed the general two-sample t-test for Chinese vs. English group in lv1, lv2, lv3, and lv4, respectively. Additionally, a 2 by 4 ANOVA was performed to examine the main effect and interaction of language (English versus Chinese) and kinship level (lv1-4). After we found the brain regions showing significant interaction between these two factors (language and kinship level) (*P*_FWE_ > 0.005, *k* > 30), we conducted region-of-interest (ROI) analyses based on the peak voxels in the language by kinship level effect. Six ROIs (left middle frontal gyrus (MFG): −47, 25, 29; left medial frontal gyrus (MeFG): −6, 19, 48; right inferior frontal gyrus (IFG): 53, 6,34; left superior parietal lobule (SPL): −25, −63, 48; right SFG: 28, −6, 58 and right precuneus (16, −75, 53) were drawn from the peak voxel in the activated clusters of language by level ANOVA. For each subject, the time series of all ROIs were extracted in a sphere region (radius = 10 mm) by MarsBar (http://marsbar.sourceforge.net/). The average parameter estimates over voxels in each ROI for each participant were tested using a 2 by 4 analysis of variance (ANOVA) followed by a post hoc Bonferroni correction.

For the ROI-ROI functional connectivity analysis, six ROIs were derived from the significant cluster in the 2 by 4 ANOVA. We used the SPM toolbox conn (v14, http://www.nitrc.org/projects/conn/) to perform the ROI-ROI FC analysis. Before averaging individual voxel data, the waveform of each brain voxel was filtered using a bandpass filter (0.01–0.1 Hz) to reduce the effect of low-frequency drift and high-frequency noise. After normalization to the MNI template and motion correction, bivariate correlations were calculated as a measure of strength of FC, to examine cross-correlations of BOLD signal time series between ROIs. For each individual, the fMRI time series were extracted for each ROI using Marsbar. Task-specific (four kinship levels for two groups) ROI-ROI FC maps were represented at *P* < 0.05 (FDR-corrected for multiple comparisons) with BrainNet toolbox (http://www.nitrc.org/projects/bnv/).

To control for the potentially confounding factor of differences in kinship intimacy causing differences in kinship term retrieval difficulty between two groups, we conducted a second-level 2 by 4 ANOVA with RT, intimacy ratings, and existing kinship relatives of each subject as covariates.

## Results

### Behavioral results

**Figure 2:**
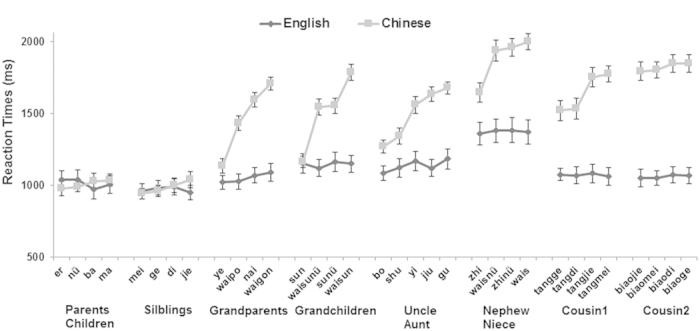
The reaction time results in four kinship levels for two groups. The dark color lines represent results of English subjects and the light color lines represent results of Chinese subjects. The error bars represent the standard error for each condition. Basically, we found significantly longer RTs for Chinese subjects in lv2, lv3 and lv4.

A language (Chinese vs. English) by level (lv1, lv2, lv3, and lv4) ANOVA indicated a significant main effect of language on reaction time, *F*_1,38_ = 30.06, *P* < 0.001, 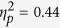. Chinese participants took longer to report the kin term (*M* = 1477.19 ms, *SE* = 48.13) than English-speaking participants (*M* = 1104.19 ms, *SE* = 48.13). Furthermore, we found significant effects of kinship level, *F*_3,114_ = 177.74, *P* < 0.001, 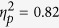, indicating that RT increased with the four kinship levels. The significant language by kinship level interaction (*F*_3,114_ = 75.80, *P* < 0.001, 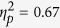) suggested that Chinese versus English group differences were observed for lv2, lv3, and lv4, but not for lv1. Chinese participants had slower responses than English participants in all three levels, all *P* < 0.001 (see Fig. 2), except lv1 (*P* = 0.95).

We found a common kinship level effect and large differences in intimacy ratings across the Chinese and English subject groups (see supplemental materials). Specifically, a significant main effect of kinship level (*F*_1,38_ = 74.09*, P* < 0.001, 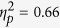) indicated closer intimacy with increasing kinship level. Additionally, the Language by kinship level interaction effect was nearly significant (*F*_1,38_ = 2.55*, P* = 0.06, 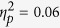), as Chinese subjects had higher intimacy ratings for lv1, lv2, and lv4 kin (all *P* < 0.05).

### fMRI results

#### Overall activation and common kinship effect

**Figure 3:**
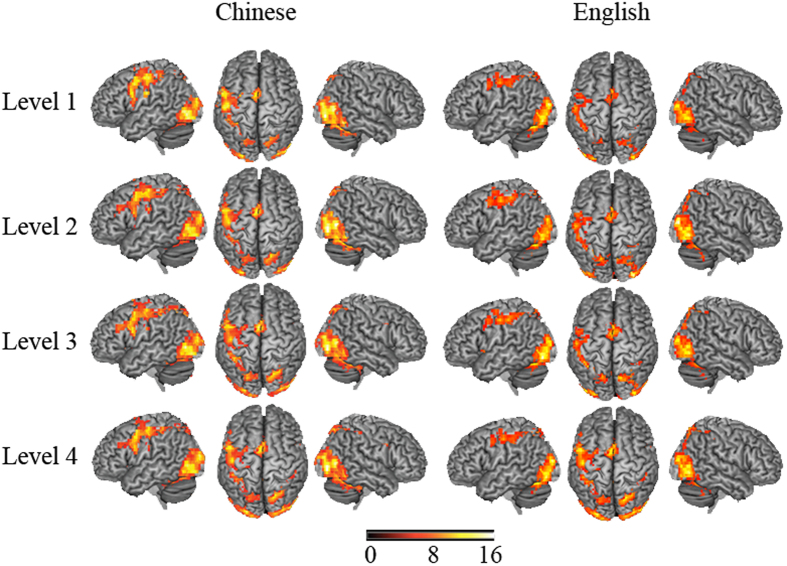
Brain activation in the kinship identification task for two groups. Activations for all the four kinship levels in Chinese subjects are shown in the left panel, while activations for all the four kinship levels in English subjects are shown in the right panel. The statistical maps show common BOLD activity over fronto-parietal cortex for two groups during kinship task, especially in the left hemisphere. Images are shown with a statistical threshold of FWE corrected *P* < 0.005, *k* > 30. Coordinates and statistics are provided in Table 1.

(a) Overall activation: Our fMRI analysis of Chinese participants revealed several brain regions that were commonly and differentially activated by different levels of kinship (Fig. 3 and Table 1). Overall, a wide range of activity in the fusiform gyrus, thalamus, and inferior and middle occipital gyri (IOG and MOG) was found for all four levels. The IFG (BA9) was consistently activated across all four kinship levels. In contrast, lv1 and lv2 significantly activated the MeFG (BA6) around the midline of the brain. For lv1 kinship, the left precentral gyrus was also activated in Chinese subjects.

English-speaking participants showed a similarly wide range of occipital, frontal, and parietal activation (Fig. 3 and Table 1). Specifically, the IOG, MOG, postcentral gyrus, MFG, and SPL were consistently activated for all levels of kinship. In addition, the SFG was also activated for lv2, lv3, and lv4 kinship, and the dorsal anterior cingulate area (BA32) was activated for lv3 and lv4 kinship.

**Figure 4:**
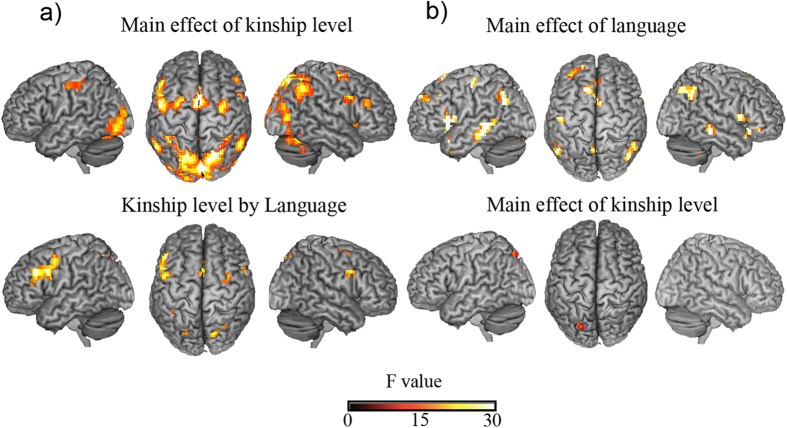
(**a**) Significant activation in language by kinship level ANOVA without covariates. We found significant main effect of kinship level over fronto-parietal cortex, and the language by kinship level effect also illustrated in left side. Thresholds of activation maps are set at *P*_FWE_ < 0.005 with a minimum cluster extent of 30 voxels. (**b**) Significant activation in language by kinship level ANCOVA, in which with RTs, intimacy rating and existing kinship as covariates. The significant main effect of kinship level or language effect is illustrated in yellow color. We find the SPL activity in the main effect of kinship level after task difficulty, intimacy and existing kinship were controlled. Thresholds of activation maps are set at *P*_FWE_ < 0.05 with a minimum cluster extent of 20 voxels.

(b) The main effect of kinship in language by kinship ANOVA: Such common activation was confirmed in the language by kinship level ANOVA. We identified a main effect of kinship level over a number of cortical regions in the frontal, occipital, and parietal cortices (see Fig. 4a and Table 2), such as the MOG, precentral gyrus, precuneus, IPL, MeFG, and IFG. Generally, activation over such brain regions was greater with increased kinship levels.

(c) ANCOVA results revealing main effects of kinship: With RT, intimacy rating, and existing kinship relative numbers as covariates, the 2 by 4 ANCOVA revealed significant effect for kinship level over the superior parietal lobule (see Fig. 4b), indicating a common role of the SPL in kinship level processing between the two groups. In this instance, the language by kinship level interaction effect showed no supra-threshold activity.

#### Chinese versus English-speaking comparison

**Figure 5:**
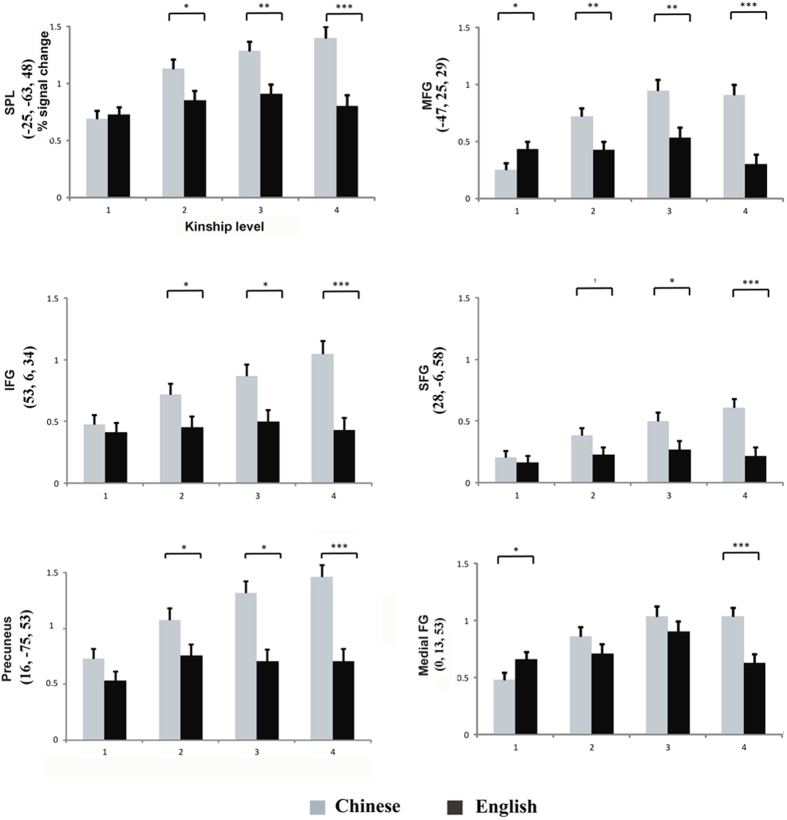
Mean parameter estimates in the six ROIs identified from the ANOVA analyses. Overall, Chinese subjects showed stronger activation in SPL, Precuneus, IFG, SFG, MFG and MeFG for higher kinship levels. Notably, English subjects showed greater activity over MFG and MeFG for the first kinship level. ^†^0.05 < *P* < 0.1, *P < 0.05, **P < 0.01, ***P < 0.001.

(a) Two sample t-test for different kinship levels: Table 3 shows all significant areas of activation observed in the Chinese versus English comparison. Chinese participants showed greater activation than English-speaking participants for lv3 and lv4, but not for lv1 and lv2. More specifically, the lv3 kinship task recruited greater activity in the frontal cortex (i.e., the MFG, IFG and SFG), precuneus, MOG, thalamus, and SPL. Compared with English-speaking participants, Chinese participants consistently showed greater activation in the frontal, parietal, and occipital cortices for the lv4 kinship task, which may be explained by the involvement of working memory in kinship inference.

(b) Interaction between language and kinship and the ROI analysis results: Significant interactions were observed between language and kinship level over a number of brain regions such as the MeFG, MFG, SFG, and regions within the parietal cortex. To systemically investigate the interaction between language and kinship level, the percent signal changes of six ROIs were included in the language by kinship level ANOVA; the post hoc analysis results are shown in Fig. 5. All of these ROIs displayed a significant interaction effect of language by kinship level, with language differences generally being more pronounced for higher kinship levels (lv3 and lv4).

Specifically, over the SPL, the significant interaction effect (*F*_3,114_ = 30.97, *P* < 0.001, 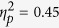) indicated that enhanced activation occurred in Chinese subjects for lv2, lv3, and lv4 (see Fig. 5).

Within the precuneus, the language by kinship level interaction effect (*F*_3,114_ = 23.97*, P* < 0.001, 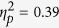) confirmed there was higher signal change in Chinese participants for lv2, lv3, and lv4 (all *P* < 0.029). Signal change over IFG also confirmed stronger activation for Chinese subjects in lv2, lv3, and lv4 (all *P* < 0.04).

Interestingly, the interaction of language by kinship level in MFG (*F*_3,114_ = 36.85*, P* < 0.001, 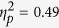) indicated that English-speaking subjects had more activation in lv1, while Chinese subjects showed higher activation for higher kinship levels. However, in the SFG, the post hoc analyses of the interaction effect (*F*_3,114_ = 51.35, *P* < 0.001, 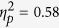) demonstrated that the language difference was nearly significant in lv2 (*P* = 0.064) and significant in lv3 and lv4, such that Chinese subjects exhibited higher SFG activation. In the MeFG, the significant language by kinship level interaction effect (*F*_3,114_ = 27.55*, P* < 0.001, 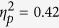) also revealed higher activation for English-speaking subjects in lv1, while Chinese subjects showed stronger activation in lv4 (all *P* < 0.05).

(c) ANCOVA results revealing main effects of language: The 2 by 4 ANCOVA revealed significant main effects of language over the superior temporal gyrus, middle temporal gyrus, parahippocampal gyrus, inferior frontal gyrus, medial frontal gyrus, and inferior parietal lobule. It showed relative stronger activity over such areas in Chinese participants.

#### ROI-ROI functional connectivity

**Figure 6:**
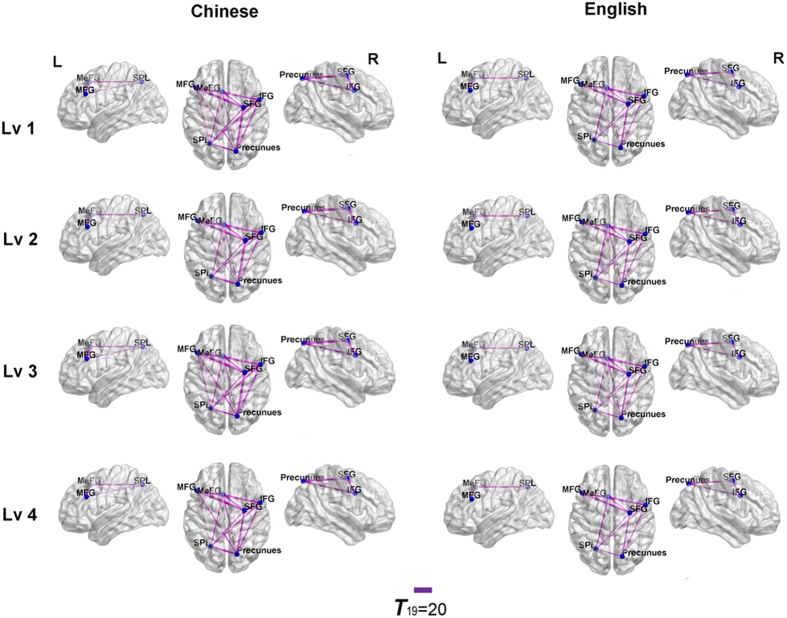
The ROI-ROI functional connectivity map for two groups. Task-specific ROI-ROI FC maps were represented at P < 0.05 (FDR-corrected for multiple comparisons) and the purple lines represent the positive correlation between two connected ROIs. We observed a common closely connected frontal-parietal network in both groups during the FEKI task. However, English subjects did not have a connection between the left MFG and left SPL (from lv1 to lv4) or between the left MFG and right precuneus (lv1, lv2, and lv4).

Functional connectivity analysis between the six ROIs indicated common and different connectivity patterns in the two groups (see Fig. 6). In all four kinship conditions in the two groups, connectivities among prefrontal brain regions and parietal regions (e.g., the IFG, SFG, MeFG, SPL, and precuneus) were observed, indicating a common closely connected frontal-parietal network in both groups during the kinship identification task. Unlike Chinese subjects, English subjects did not have a connection between the left MFG and left SPL (from lv1 to lv4) or between the left MFG and right precuneus (lv1, lv2, and lv4).

#### Correlation with behavioral results

After the ROI analyses, we obtained the mean parameter estimates in six ROIs for each participant. We were concerned about the relationship between the activities of these brain regions and the individual’s RT. Therefore, we performed six separate correlation analyses, but only found a significant positive correlation between MFG activation and RTs (*r* = 0.424, *P* < 0.01). Specifically, subjects with higher MFG activity had longer RTs. However, we did not find any significant correlation between intimacy rating and activation of the six ROIs (all *P* values > 0.10).

## Discussion

To date, most neuroscience research on kinship has focused on the neural basis of identifying specific kinships from external cues. Few studies have investigated the representations of kinship levels in different kin term systems. We attempted to explore cross-language differences in kinship terms representation from a cognitive neuroscience point of view by asking native Chinese and English speakers, for whom the kin term systems are markedly different, to perform a kinship identification task. As we hypothesized, we observed common brain architecture reflecting kinship structure along with variations that reflected the differences in kinship classification constraints such as parental lineage, relative age, and other factors (Kroeber, 1933; Watson, 1982; Dos Santos, 2006).

Indicative of common kinship level effects on RT and brain activation, we found that RTs and frontal-parietal brain activation increased with kinship levels; this effect is in agreement with the findings of our previous behavioral study that only examined Chinese subjects^18^. Therefore, fMRI results and the functional connectivity analysis showed kinship representation recruits a common fronto-parietal brain networks in both groups. Interestingly, these brain regions were also implicated in kinship level effects observed in specific areas including the IFG, MFG, SPL, and IPL, especially in lv1 to lv3. Main effects of kinship level indicating that the fronto-parietal network was more responsive to higher kinship level were confirmed in ROI analysis (see Fig. 5). The FEKI task requires subjects to compare location and generation between a prime unit and a target unit. Therefore, we postulate that the frontal-parietal activation pattern we observed during this task reflected attention control and distance or grade processing. Previous studies that used similar cue-target tasks have also reported comparable frontal-parietal cortex activation^20^,^21^, which has been interpreted to be indicative of attention control^22^,^23^.

To summarise, the results of our current study are consistent with the kinship level effect we previously observed in Chinese subjects and reveal a common fronto-parietal brain network responsible for kinship representation. Although there were target number differences between the kinship levels, we argue that our results cannot be attributed to such differences alone, because the number of black targets in the kinship space was the same for lv2 and lv3, while lv4 had the most targets. However, we found a stable main effect of kinship level on RT and brain activity between lv2 and lv3 (even though the ROI results showed differences).

More importantly, our results revealed significant differences across the two groups in RT and brain activation, especially for higher kinship levels (lv3 and lv4). For example, Chinese subjects showed longer RTs than English-speaking subjects in lv2, lv3, and lv4. Moreover, two-sample t-tests showed that Chinese subjects had greater activities in the SPL, MFG, and IFG for lv3 and lv4, which may reflect a difference in kinship representations between the two languages. In the kinship space, all possible terms were determined by constraints such as sex, distance, patrikin, generations, and age. As stated above, there are more constraints for Chinese subjects because the kinship inference involves more rules at higher kinship levels. For example, in English, cues of “mother’s brother and father’s brother” would elicit the response of “uncle”, regardless of the uncle’s age. However, in Chinese, a cue of “brother, younger or older than the father” would represent “Shu1Shu1 or Bo2Fu4”, respectively (see Table S1). Additionally, the corresponding kinship term is distinct for the mother’s brother. Therefore, Chinese subjects need to consider more rules for higher kinship levels, such as patrikin and age, which may account for the longer RTs we observed in the Chinese group.

Our explanation is also in line with the assumption of serial filtering processes of kinship constraints in the optimality theory^24^. From this perspective, the RTs also validate that our paradigm could differentiate the kinship structure defined by various kin classification rules in diverse cultures. The current results suggest that six brain regions (the MFG, IFG, SFG, MeFG, and SPL/precuneus) over the frontal and parietal cortices exhibit language differences and that Chinese subjects generally show greater activities over these regions for higher kinship levels. Given that there are more kinship constraints in the Chinese kinship classification, it is reasonable to hypothesize that higher activity is associated with greater attention control, number magnitude, or distance processing.

According to the number processing model proposed by Dehaene^25^,^26^, the bilateral parietal lobes, especially the intraparietal sulcus and IPL, mediate mental representations of number magnitude. With respect to the current results, IPL activity in both groups, which was affected by kinship level, may be responsible for determining kinship distance or the grade of kinship, which is consistent with previous social class studies showing IPL activation^27^,^28^,^29^. For instance, Chiao *et al.*^30^ found that the IPL was activated in both social class and number comparison tasks, and that comparisons of short distances elicited greater activity than comparisons of long distances. However, our current results indicated that IPL activity was greater in longer distance kinship levels, which may be due to differences in task difficulty. In the current kinship identification task, long-distance kinship was the highest level of difficulty that subjects encountered; thus, compared to distance comparisons studied by Chiao *et al.*^30^, our task was relatively more difficult. A recent study showed common neural mechanisms of spatial and social distance in the IPL^31^, both of which are reflected in our task. Because the social status of a family member is strictly linked to generation, sex, and age in the kinship space in a traditional Chinese family^32^,^33^,^34^, such cultural dissimilarities in IPL activation may be related to differences in social rank processing.

Considering these previous findings, we suggest that the IPL activity observed in the present study was associated with the representation of kinship level or social distance. In contrast, although the posterior parietal cortex, including the SPL, is also activated in tasks of number processing such as number comparison^35^,^36^, estimate calculation^37^, double-digit subtraction^38^, and counting^39^, the SPL and precuneus activities observed in our study could be interpreted to be a manifestation of spatial attention in the kinship space task. According to the three parietal circuits for number processing model proposed by Dehaene *et al.*^25^, the SPL is not a number-specific brain region; rather it is associated with the attention process. Moreover, the SPL is consistently activated when top-down attention is required, such as in spatial attention tasks^40^,^41^,^42^,^43^,^44^,^45^,^46^, crossmodal attention shifts^47^,^48^,^49^,^50^, and spatial working memory^51^,^52^,^53^. Transcranial magnetic stimulation (TMS)^54^,^55^ and brain lesion^56^ studies have also provided evidence of SPL activation in visuospatial functions. We found that the SPL was sensitive to kinship level in both Chinese and English, even when controlling for task difficulty and intimacy. We propose that the stronger SPL or precuneus activity in higher kinship levels (lv2, lv3, and lv4) was associated with the greater spatial attention or spatial working memory load required for identifying kinships. Specifically, identifying higher kinship levels involves more spatial attention by combining multiple kinship agents in the kinship space.

Considering the correlation between RT and activation in MFG and the disappearance of the language by kinship level effect after controlling for RT, intimacy, and existing kinship, we believe that the activation in the PFC was mainly associated with task difficulty-related working memory and attention control. The role of the PFC in top-down attention control has been well established in previous studies^57^,^58^,^59^. In the current task, higher kinship levels were represented by more targets in the kinship space. To selectively inhibit the key-press before reporting the farthest target, subjects needed to exert top-down control processing, which recruited prefrontal brain regions such as the MFG, SFG, and IFG. Therefore, task difficulty may account for the greater PFC activity that we observed in Chinese individuals (compared to English subjects) at higher kinship levels, especially for lv4. Considering that the MFG, IFG and SPL are related to the success of spatial working memory^60^, the weakened or absent functional connectivity between the MFG and SPL/precuneus in English-speaking subjects may also be attributable to the reduced spatial attention and working memory load required to identify kinships in English, given that they contain less constraints and thus require less spatial location cues (e.g., higher/older or lower/younger).

The current study has several noteworthy limitations. First, task difficulty may account for some of the differential effects observed between the two groups. However, this does not explain the finding that for lv1 English-speaking subjects showed greater MPFC activation than Chinese subjects in absence of RT differences. Our results thus at least reflect the universal kinship constraints (e.g., generation) and the different kinship constraints (e.g., relative age) present across languages. Secondly, the sample size may limit our conclusions, especially given the huge population of Chinese and English speakers, further cross-cultural studies with greater sample size thus could help confirm our results.

Taken together, our study is the first to report common kinship terms representations reflected in both behavioral and neuroimaging studies in two different languages, suggesting that common neural substrates in the frontal-parietal cortex are recruited for kinship identification tasks. Given our previous finding that kinship was analogous to the spatial representation of the physical space^24^, our results suggest that the kinship space may be analogous to activation patterns of a frontal-parietal spatial representation brain network. Moreover, we found striking group differences in the RT and fMRI results, especially at higher kinship levels. Overall, Chinese subjects exhibited longer RTs and greater frontal-parietal activation due to their more complex kinship classification system. As the first study investigating language differences in kinship identification, our results advance our understanding of how both the similar and different representation of kinship terms between two languages.

## Additional Information

**How to cite this article**: Wu, H. *et al.* Language modulates brain activity underlying representation of kinship terms. *Sci. Rep.* **5**, 18473; doi: 10.1038/srep18473 (2015).

## Supplementary Material

Supplementary Information

## Figures and Tables

**Table 1:** Peak activated brain regions for the four levels kinship in Chinese and English participants (*P* < 0.005, FWE-Corrected, *k* > 30 voxels).

Region	Chinese							English				
	Side	BA	Peak t-value	x	y	z		BA	Peak t-value	x	y	z
Level 1												
Inferior Temporal Gyrus	R	20	17.07	50	−53	−14						
Inferior Occipital Gyrus	L	18	14.28	−41	−78	−5	L	19	14.13	−41	−81	−10
	R						R	19	13.35	41	−75	−5
Precuneus	R	7	9.33	28	−72	38	R	7	10.82	28	−66	38
Superior Parietal Lobule	R	7	9.41	19	−69	58	L	7	10.08	−25	−59	48
Inferior Parietal Lobule	L	40	10.62	−31	−47	53	L	40	9.72	−34	−47	48
Precentral Gyrus	L	3	13.6	−56	−16	29						
Medial Frontal Gyrus	L	6	11.85	−3	0	62						
Superior Frontal Gyrus							R	6	10.72	3	9	58
Postcentral Gyrus							L	3	9.82	−56	−16	48
Level 2												
Fusiform Gyrus	R	19	15.93	31	−63	−19						
Middle Occipital Gyrus	R	19	15.34	41	−85	−5	R	19	16.2	31	−88	14
	L		14.38	−44	−69	−10	L	37	15.58	−41	−72	0
Precuneus	R						R	7	13.12	28	−66	38
Superior Parietal Lobule	L	7	14.59	−28	−59	48	L	7	12.17	−25	−59	58
Medial Frontal Gyrus	L	6	12.98	−3	0	67	L	6	11.54	−3	−3	62
Putamen	L		12.37	−25	−3	5						
Thalamus							L		12.53	−19	−22	5
Level 3												
Inferior Occipital Gyrus	L	18	13.44	−38	−85	−10	L	19	16.03	−38	−81	−10
	R						R	19	14.3	41	−72	−5
Middle Occipital Gyrus	R	19	17.38	50	−56	−10	R	19	12.53	47	−75	5
	L	19	14.85	−47	−75	0	L	37	15.73	−41	−72	0
Medial Frontal Gyrus	L	6	10.33	−6	13	48	L	6	12.23	−3	0	62
Superior Frontal Gyrus	L	6	14.03	−3	3	67	R	6	11.56	3	9	53
Postcentral Gyrus							L	2	11.69	−41	−28	43
Putamen	L		16.29	−22	−3	5	R		12.09	22	−3	−5
Precunues							R		11.74	28	−66	38
Thalamus							L		11.45	−19	−22	10
Level 4												
Inferior Occipital Gyrus	R	18	16.03	41	−85	−5	R	19	15.85	41	−72	−5
Thalamus			15.84	28	−31	5	L		14.43	−16	−22	0
Superior Parietal Lobule	L	7	12.26	−31	−53	53	R	7	12.59	28	−66	48
Superior Frontal Gyrus	L	6	12.4	−3	3	67	R	6	10.52	0	9	53
Middle Occipital Gyrus	L	37	15.19	−44	−69	−10						
Inferior Frontal Gyrus	L	9	15.08	−47	6	34	L	9	7.98	−44	3	34
Precuneus	R	7	11.22	28	−72	34	L	7	12.18	−25	−63	53

Note. BA refers to putative Brodmann’s Area; L and R refer to left and right hemispheres; x, y, and z refer to MNI coordinates in the left–right, anterior–posterior, and inferior–superior dimensions, respectively; t refers to the t-score at those coordinates (local maxima).

**Table 2:** Peak activation in language by kinship level ANOVA (FWE corrected, *p* < 0.005, *k* > 30 voxel).

Brain region		BA	F	x	y	z
Main effect of kinship level						
Cuneus	R	19	59.91	3	−85	29
Medial Frontal Gyrus	R	6	52.06	0	13	53
Middle Frontal Gyrus	R	6	33.45	31	−3	58
Inferior Parietal Lobule	R	40	25.86	56	−53	48
	L	40	30.68	−59	−56	38
Thalamus	L		29.8	−6	−19	14
Inferior Frontal Gyrus	R	9	24.61	50	6	34
Language by kinship level interaction						
Middle Frontal Gyrus	L	46	30.46	−47	25	29
Inferior Frontal Gyrus	R	9	23.78	53	6	34
Superior Parietal Lobule	L	7	30.10	−25	−63	48
Cingulate Gyrus,/medialFG	L	32	23.71	−6	19	48
Precuneus	R	7	20.43	16	−75	53
Superior Frontal Gyrus	R	30	18.8	28	−6	58

**Table 3:** Significantly activated brain regions in two sample t test (*P* < 0.05, FDR-Corrected, *k* > 30 voxels).

Brain region		*BA*	*T*	x	y	*z*
Level 3(Chinese > English)						
Precuneus	L	7	5.56	−22	−78	53
Precentral Gyrus	R	6	4.82	31	6	34
Superior Parietal Lobule	R	7	4.78	31	−53	43
Middle Occipital Gyrus	L	19	4.7	−31	−81	10
Middle Frontal Gyrus	R	6	4.15	31	−9	62
Inferior Frontal Gyrus	R	47	4.45	28	22	14
Putamen	L		4.42	−28	−19	−10
Caudate	L		3.79	−13	19	10
Level 4(Chinese > English)						
Precuneus	L	7	7.01	−22	−75	53
Inferior Frontal Gyrus	R	9	6.49	47	9	29
Middle Frontal Gyrus	L	46	5.39	−47	25	34
	R	6	4.28	31	9	53
Middle Temporal Gyrus	R	39	4.65	53	−72	19
