# Supplementary material for: Language modulates brain activity underlying representation of kinship terms
Source: Sci Rep. 2015 Dec 21;5:18473. doi: 10.1038/srep18473 (PMC4685275; doi:10.1038/srep18473)
Supplement: Supplementary Information [file srep18473-s1.doc]

Language modulates brain activity underlying representation of kinship terms

Haiyan Wu, Yue Ge, Honghong Tang, Yue-Jia Luo, Xiaoqin Mai, Chao Liu*

**Table S1.** Chinese kin terms (without affines)

| **Level** | **Chinese** | **English** | **Relation to Ego** |
| --- | --- | --- | --- |
| **1** | ba | father | Father |
|  | ma | mother | Mother |
|  | er | son | Son |
|  | nü | daughter | Daughter |
|  | ge | brother | Older brother |
|  | di |  | Younger brother |
|  | jie | sister | Older sister |
|  | mei |  | Younger sister |
|  |  |  |  |
| **2** | ye | grandpa | Father’s father |
|  | lao.ye |  | Mother’s father |
|  | nai | grandma | Father’s mother |
|  | lao |  | Mother’s mother |
|  | sun | grandson | Son’s son |
|  | wai.sun |  | Daughter’s son |
|  | sun. nü | granddaughter | Son’s daughter |
|  | wai.sun.nü |  | Daughter’s daughter |
|  |  |  |  |
| **3** | shu | uncle | Father’s Younger brother |
|  | bo |  | Father’s Older brother |
|  | jiu |  | Mother’s brother |
|  | gu | aunt | Father’s sister |
|  | yi |  | Mother’s sister |
|  | zhi | nephew | Brother’s son |
|  | wai.sheng |  | Sister’s son |
|  | zhi. nü | niece | Brother’s daughter |
|  | wai.sheng. nü |  | Sister’s daughter |
|  |  |  |  |
| **4** | tang.ge | cousin | Father’s brother’s ***son*** that is ***older***  than Ego |
|  | tang.jie |  | Father’s brother’s ***daughter*** that is ***older*** than Ego |
|  | tang.di |  | Father’s brother’s ***son*** that is ***Younger*** than Ego |
|  | tang.mei |  | Father’s brother’s ***daughter*** that is ***Younger*** than Ego |
|  | biao.ge |  | Father’s sister’s or mother’s brother & sister’s ***son*** that is ***older*** than Ego |
|  | biao.jie |  | Father’s sister’s or mother’s brother & sister’s ***daughter*** that is ***older*** than Ego |
|  | biao.di |  | Father’s sister’s or mother’s brother & sister’s ***son*** that is ***Younger*** than Ego |
|  | biao.mei |  | Father’s sister’s or mother’s brother & sister’s ***daughter*** that is ***Younger*** than Ego |

Meaning of the prefix and suffix: wai-“outside”; nü- “female”; tang- “father’s brother’s ”; biao- “father’s sister’s or mother’s brother & sister’s”

**Figure S1**


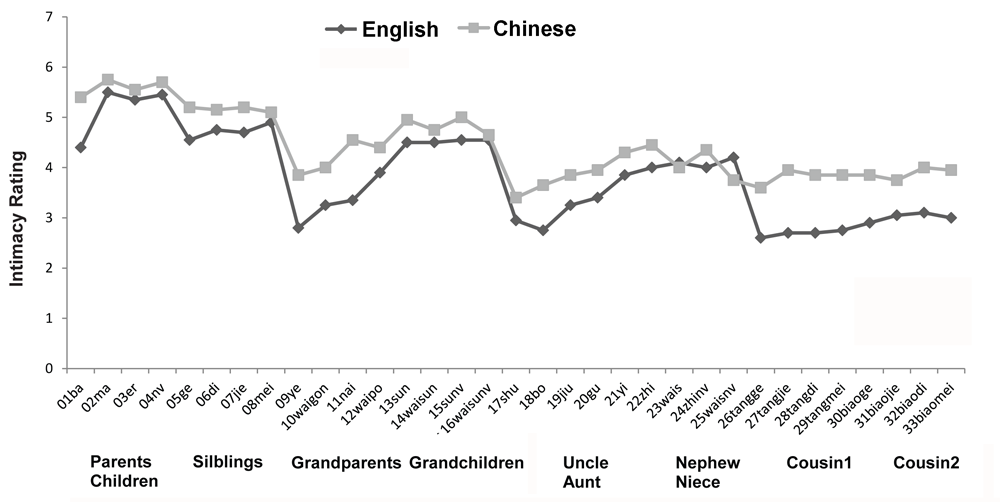


Intimacy ratings across two languages
